# SNPs and Other Features as They Predispose to Complex Disease: Genome-Wide Predictive Analysis of a Quantitative Phenotype for Hypertension

**DOI:** 10.1371/journal.pone.0027891

**Published:** 2011-11-30

**Authors:** Joong-Ho Won, Georg Ehret, Aravinda Chakravarti, Richard A. Olshen

**Affiliations:** 1 VA Cooperative Studies Program, Mountain View, California, United States of America; 2 Division of Biostatistics, Stanford University, Stanford, California, United States of America; 3 McKusick-Nathans Institute of Genetic Medicine, Johns Hopkins University, Baltimore, Maryland, United States of America; 4 Division of Cardiology, Geneva University Hospital, Geneva, Switzerland; 5 Department of Epidemiology and Medicine, Johns Hopkins University, Baltimore, Maryland, United States of America; Naval Research Laboratory, United States of America

## Abstract

Though recently they have fallen into some disrepute, genome-wide association studies (GWAS) have been formulated and applied to understanding essential hypertension. The principal goal here is to use data gathered in a GWAS to gauge the extent to which SNPs and their interactions with other features can be combined to predict mean arterial blood pressure (MAP) in 3138 pre-menopausal and naturally post-menopausal white women. More precisely, we quantify the extent to which data as described permit prediction of MAP beyond what is possible from traditional risk factors such as blood cholesterol levels and glucose levels. Of course, these traditional risk factors are genetic, though typically not explicitly so. In all, there were 44 such risk factors/clinical variables measured and 377,790 single nucleotide polymorphisms (SNPs) genotyped. Data for women we studied are from first visit measurements taken as part of the Atherosclerotic Risk in Communities (ARIC) study. We begin by assessing non-SNP features in their abilities to predict MAP, employing a novel regression technique with two stages, first the discovery of main effects and next discovery of their interactions. The long list of SNPs genotyped is reduced to a manageable list for combining with non-SNP features in prediction. We adapted Efron's local false discovery rate to produce this reduced list. Selected non-SNP and SNP features and their interactions are used to predict MAP using adaptive linear regression. We quantify quality of prediction by an estimated coefficient of determination (*R* ^2^). We compare the accuracy of prediction with and without information from SNPs.

## Introduction

Persistent idiopathic elevated blood pressure (BP), or essential hypertension (HTN), is quantitatively the major risk factor for untoward cardiovascular outcomes, with wide-ranging prevalence of 29% in the U.S. Although pathogenic pathways that lead to HTN remain poorly understood, much of the risk of HTN is believed to be genetic. Therefore, genetic investigations may lead to our improved understanding of traits related to HTN and ultimately to identifying new molecular targets for drug therapy. Genome-wide association studies (GWAS) are the most recent form of such efforts and have been employed to interrogate the genetic architecture of other complex diseases as well as essential HTN [1]. The number of susceptibility variants that can be identified definitively by GWAS is limited although they can be used to gain insights into disease pathways [2], [3], [4]. Since for genetically complex disease its heritability caused by mutation away from wildtype in any single SNP seems quite small, contributions of these susceptibility SNPs, if any, to prediction of complex traits of interest is limited. Indeed, as we show in this article, at least for mean arterial BP, SNPs do not add much and sometimes diminish, predictive information available from other clinical features.

From a clinical point of view, we are interested in how much new information the genome-wide SNPs provide in prediction of a BP trait in addition to what is achievable from traditional risk factors, such as blood cholesterol level and glucose level. To this end, population-based cohort studies that include rich data on non-SNP clinical biomarkers, if combined with whole-genome SNP data, could complement case-control studies such as GWAS in a clinically relevant fashion. This approach shifts the focus of studies from finding genes that are causally associated with disease status (which can be understood as multiple hypothesis testing with very stringent type I error rate imposed in order to avoid false positives due to the vast number of hypotheses) toward assessing predictive power for the unobserved phenotype of interest, which can be modeled by regression with many predictors.

In this article we quantify the impact on mean arterial blood pressure (MAP), an obvious quantitative phenotype, of more common genetic variation above and beyond that of other conventional risk factors (non-SNP clinical features), in the cohort of first-visit Atherosclerosis Risk in Communities (ARIC) women. ARIC was a prospective study, conducted in four U.S. communities, supported by the U.S. National Heart, Lung and Blood Institute (NHLBI). What makes the ARIC cohort an ideal study sample is that there were many measurements gathered in clinic, not least blood pressure measured according to the ARIC protocol [5]. We chose MAP for the quantitative phenotype of interest. MAP is one of the four major blood pressure components; systolic (SBP) and diastolic blood pressure (DBP), pulse pressure, and MAP. Each has been shown to be associated with cardiovascular risk, all the better if used in combination [6]. MAP is the average pressure in the arterial system and was chosen here rather than SBP or DBP because it represents a physiological (rather than traditional) component of blood pressure, corresponding to the product of cardiac output and peripheral resistance minus central venous pressure [6]. MAP is highly correlated with SBP and DBP (squared correlation coefficients *r* ^2^ of 0.81 and 0.84 in our study sample, consistent with what has been described by others [7]). Although the usefulness of MAP as an independent cardiovascular risk factor beyond SBP and DBP is debated, we would expect very similar results if SBP or DBP were used in our analysis. MAP can be estimated by a convex combination of systolic (SBP) and diastolic blood pressure (DBP): 2/3 DBP+1/3 SBP, a simplification that we take as our definition. Also, MAP as opposed to hypertensive status can be measured, or at least approximated, non-invasively without the biases related to clinically diagnosed cases and controls, allowing for subtle phenotypic variability among individuals. Potential genetic determinants of brachial SBP and DBP were evaluated in several prior studies [8], [9], [10], [11], but separate consideration of MAP has not been investigated fully.

We consider the synergistic impacts of SNPs and other features with initial hypothesis that variation in MAP can be explained largely by non-SNP features together with SNPs, and the synergistic effects of their union. To begin, we assess the ability of non-SNP features to predict MAP for white female subjects. Predictive analysis of SNPs on MAP is conducted with a novel regression technique that has two stages: (a) “main effects” and “interactions” are discovered for non-SNP features by applying classification and regression trees (CART) combined with the bootstrap for which the sampling units are individual subjects; this selection of non-SNP features and their interactions enables assessment of accuracy in predicting MAP; (b) the enormous list of SNPs genotyped is pared down to a manageable list for the purpose of combining with non-SNP features in prediction; to that end, we adapt the local false discovery rate (*locfdr*) framework [12], [13] in order to find main effects of SNPs as they bear upon prediction; we employ then the selected non-SNP and SNP features to predict MAP using adaptive linear regression. The crucial rationale here is that a SNP is unlikely to have a synergistic effect if it shows no individual predictive power. The predictive power of SNPs and subsequently their synergistic effects are quantified by an estimated coefficient of determination (*R^2^*). This number is compared with *R^2^* estimated from stage (a) in order to quantify improvement in prediction due to SNPs. We validate the entire procedure by 10-fold cross-validation.

As stated, the purpose of the proposed predictive modeling is to assess whether knowledge of genetic variants can improve the accuracy of prediction, as measured by *R^2^* of MAP above and beyond that obtained with conventional risk factors. Note that this is a slightly different goal from that of typical association studies in the sense that the selected SNPs need not meet stringent levels of significance for multiple hypothesis testing. Rather, this addresses the question of which variants are most predictive of MAP, as highly associated SNPs are not always good predictors of the phenotype [14]. Hence, cross-validated discovery of the relationship between genome-wide SNPs and the phenotype of interest is relevant. For this, model selection approaches are required to find the set of SNPs that best predicts the phenotype [15]. Model selection is handled by incorporating the adaptive regression framework.

## Methods

All individuals provided written informed consent, including consent for genetic studies; this research was approved by The Office of Human Subjects Research Institutional Review Boards of Johns Hopkins University, Committee JHM-IRB 2 on December 2, 2010. In addition, the research proposed in the ARIC study including research done for this ancillary study has been carried out according to guidelines expressed in the Helsinki Declaration.

### Study samples

All research was completed with written informed consent and the data were analyzed anonymously. All clinical investigation was conducted according to the principles expressed in the Declaration of Helsinki.

Among the 8861 individuals, all of whom are self-reported whites, genotyped among the 15,792 ARIC individuals, we selected 3138 females who were premenopausal or naturally postmenopausal at the first visit. We concentrate on women rather than men because the genetics of what drives blood pressure is different in men than women. We focus on pre-menopausal and naturally post-menopausal women because we do not want medical interventions to interfere with the joint relationship of predictors and outcome. For each individual, we used 377,790 unimputed SNPs on the autosomal chromosomes. As non-SNP clinical features, we used 44 variables that include morphological and biomarker measurements. The 44 clinical features were chosen subjectively because it was thought that they would be most predictive among available ARIC features. Readers can see what features were available from ARIC (http://www.cscc.unc.edu/aric/). The full list and the characteristics of these variables for the sample of subjects included in our analyses are presented in Table S1. In particular, age at the first visit took values from 44 to 66, with mean 54.84. Each feature had missing values for at most 2% of people. We imputed missing values in these non-SNP features using CART trained on the known values as responses and the other features as predictors, following [16].

The mean and standard deviation of observed (and treatment corrected; often in computations of blood pressure, there is an adjustment that consists of adding 10 mmHg to SBP and 5 mmHg to DBP for those on anti-hypertensive medication(s) [15]) SBP in our sample were 117.6 (120.2) and 17.82 (19.41); for DBP they were 69.76 (71.06) and 9.688 (10.29). The proportion of individuals taking anti-hypertensive medication was 26.0%.

### Model

We employed an additive model in which the phenotype of interest (MAP) is a linear function of particular non-SNP features, their interactions, additive genetic effects of SNPs, and the interactions among the SNPs and the non-SNP features. The additive model can be written
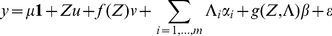
(1)where *y* is a vector of length *n* representing MAP; **1** is a vector of *n* ones; *Z* is a data matrix for the main effects of the non-SNP features; *f*(*Z*) is a data matrix for the interactions among the non-SNP features; Λ*_i_* is a column vector of length n having entries 0, 1, or 2 representing the number of minor alleles at the *i*-th SNP; and *g*(*Z*,**Λ**) is a data matrix for the interactions among the SNPs and the non-SNP features, with **Λ** = [Λ_1_, …, Λ*_m_*]. μ is the overall mean of MAP; *u* is a vector of non-SNP main effects; *v* is a vector of non-SNP interaction effects; α*_i_* is the main effect of i-th SNP; β is a vector of interactions among the SNPs and the non-SNP features; ε is a vector of residuals.

It bears mention that this paper is about variable selection and thus is in the spirit of data mining. Note that model (1) is conditional on data matrix Z, not to speak of being conditional also on available SNPs: μ is a one-dimensional constant; ν, the α's, and β are (finite dimensional but typically not one-dimensional) constants. It is fundamental that analyses given in this paper are all conditional on values of these parameters and data matrices. The source of randomness in our paper is relevant to bootstrapping discussed in the next section. Apart from the error ε, it arises from the joint empirical distribution of *Z* and Λ. This approach is in contrast with the prevailing “components of variance” approach to understanding genetic data [17], which is conditional on a model having been selected. Its most important part is inference on the additive random effects of the genetic component in a preselected model. Randomness is unconditional with respect to these effects, such as “percent variance explained.” Whatever percent this is must be taken to be in this context, but not in our framework. Issues of conditional versus unconditional bases to inference are pervasive in the statistical literature, perhaps especially in the analysis of variance and in regression. In short, variable selection for predictive modeling and estimation of a component of variance for a given model are simply not the same thing.

When model selection is the issue, as it is here, Gaussian assumptions on relevant criteria and their implications for the distribution of MSE do not apply. Therefore one way to judge “significance” is from some sort of internal validation of an entire process. The question is one of validation, not of subjective choice of predictive features or combinations of them. The remainder of Methods deals with this issue.

### Estimation of non-SNP effects

We used CART to select main effects and interactions among the 44 non-SNP features. The use of CART as an interaction selector has been advocated widely [18], although it is also well known that CART fits have large variability. To cope with potential instability, we applied the bootstrap. We sampled individuals with replacement 300 times independently. In other words, the bootstrap sampling unit is an individual. While it may be that the 3138 samples include family members, this matter was not considered in our validation because we have no information. However, it is far from obvious even if we had family structures that we would want to use them in our problem of variable selection. One can argue that randomly selected individuals in the population “out there” come with family structures. These structures are represented fairly among the ARIC data. If so, then taking account of family structures is done automatically by what we have done in our approach where the goal is selection of features.

For each bootstrap sample, we fit CART using the default pruning method. For each of the 300 trees, we chose main effects along every path from the root to the leaf node by picking a feature if it was ever the feature on which a split was made. For each path, we chose two- and three-factor interactions as *adjacent* nodes, i.e., parent-child pairs and grandparent-parent-grandchild triples. With this approach, for each interaction term the corresponding main effects are also chosen. Among the chosen main effects and the interaction terms, we selected those that occurred more than a certain percentage of the maximum 300 occurrences (we tried 1%, 5%, 10%, and 20%).

Trees have been used previously, albeit in ways different from their use here, in order to find and quantify particular interactions of amino acids at various sites of genomes. See, for example, [19], [20].

Still, features come in groups that are close to collinear. If a pair of frequent features does not occur simultaneously in a single bootstrap run of CART, they may compete due to collinearity (cf. surrogate splits; see [21], pp. 140–142). In order to find groups of competing features, we performed hierarchical clustering based on the co-occurrence matrix *D* [22] such that
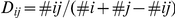
where *#ij* is the total number of simultaneous occurrences of features *i* and *j*, *#i* and *#j* are there respective number of individual occurrences. For each cluster C, we selected a centroid feature using a minimax criterion
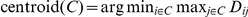
(This centroid feature was chosen from among an already existing list of features.) Using co-occurrence clustering, we obtained final non-SNP features about 40% fewer than those obtained before applying this method.

We applied the LASSO adaptive regression in order to assess the overall predictive power, possibly eliminating features with low predictive value. Note that CART is not used to directly fit MAP; it is the LASSO that is fit using the features selected by boostrapped CART and the co-occurrence matrix-based clustering. We cross-validated the entire procedure of CART, the bootstrap, the co-occurrence method, and the LASSO in order to estimate the coefficient of determination (*R* ^2^), defined as the squared correlation coefficient between the predicted responses and the actual responses. In this way, we validate the adaptive algorithm and its predictive power, but not a single model with fixed predictors.

### Estimation of main effects of SNPs

In order to assess the marginal effect of SNPs, so in addition to the predictive contribution of the non-SNP features, we computed the nominal P-value of each SNP for the model (1) with *m* = 1, β = 0 (i.e., no gene-gene or gene-environment interactions), and the covariates set to the non-SNP features chosen by bootstrapped CART and the co-occurrence clustering. SNPs that have low local false discovery rates [12], [13], a “local” empirical Bayes version of false discovery rates [23], were selected as candidate predictors in model (1), with β = 0. More precisely, we applied the step-up procedure for local false discovery rates [24] in order to bound marginal false discovery rates (mFDR). We tried the bounds 0.2 and 0.5. We validated the increase in predictive power due to the inclusion of the SNP features in the LASSO regression using the same cross-validation sets as those used for validating non-SNP features.

### Estimation of interaction effects among SNPs and non-SNP features

We explored the full model (1) by repeating the procedure of Section 2.3, but with the chosen SNPs added to the CART interaction selector. We validated the increase in predictive power of this approach using the same cross-validation sets as those used for validating non-SNP features.

## Results

### Non-SNP features alone can achieve a moderate predictive power

The adaptive prediction algorithm chose 14 to 30 non-SNP features (see Table 1), and achieved cross-validated *R* ^2^ of 24% to 27% for medication-adjusted [25] MAP and mFDR cutoff 0.2 (Table 2, “non-SNP”). For unadjusted MAP its predictive performance was lower (up to 14.6%, same mFDR cutoff; see Table 2) and the number of chosen non-SNP features ranged from 21 and 44 (Table S2). These features are typical risk factors for HTN and their interactions.

**Table 1:** Non-SNP features (above the horizontal line) and interactions (below the horizontal line) chosen by our adaptive prediction algorithm.

Code	feature	cutoff fraction of occurrence							
		0.01		0.05		0.1		0.2	
		avg coef	CVcount	avg coef	CV count	avg coef	CV count	avg coef	CV count
ANTA07A	Waist girth (cm)	2.97E−02	2	2.53E−02	4	2.88E−02	5	1.63E−02	7
APASIU01	Apolipoprotein A1 (mg/L)	NA	NA	2.53E−04	4	2.17E−04	1	NA	NA
APBSIU01	Apolipoprotein B (mg/L)	3.37E−05	3	8.38E−05	2	NA	NA	NA	NA
BMI01	Body mass index (kg/m^2^)	3.23E−02	2	1.69E−02	2	2.74E−02	1	6.15E−02	1
CENTERID.B	Field center	−3.51E−01	5	−7.11E−01	7	−2.35E−01	2	NA	NA
CENTERID.D	Field center	−1.95E+00	10	−2.18E+00	10	−1.74E+00	8	−1.31E+00	1
CHOLMD02.1	Meds that secondarily lower cholesterol	8.14E+00	10	8.45E+00	10	8.20E+00	10	7.54E+00	10
CIGT01.2	Cigarette smoking status (% never)	6.01E−01	6	8.89E−01	7	4.49E−01	4	6.28E−01	1
CIGT01.3	Cigarette smoking status (% never)	9.73E−01	10	1.26E+00	9	8.92E−01	6	8.96E−01	2
CIGTYR01	Cigarette years of smoking	−6.29E−04	9	−7.00E−04	8	−5.54E−04	6	NA	NA
ETHANL03	Usual ethanol intake (g/week)	5.60E−03	6	6.21E−03	4	2.28E−03	3	NA	NA
INSSIU01	Insulin (pmol/L)	7.81E−04	2	6.11E−04	4	3.49E−04	4	NA	NA
TCHSIU01	Total cholesterol (mmol/L)	5.84E−01	1	5.88E−01	2	5.80E−01	5	5.31E−01	10
TRGSIU01	Total triglycerides (mmol/L)	3.58E−01	5	3.82E−01	9	3.11E−01	9	1.34E−01	4
V1AGE01	Age at first visit	1.01E−01	9	1.31E−01	10	1.24E−01	10	1.03E−01	3
WSTHPR01	Waist-to-hip ratio	1.73E+00	3	1.94E+00	2	4.54E+00	2	5.61E+00	2
ANTA07A:TCHSIU01		8.10E−03	5	7.32E−03	4	8.17E−03	1	NA	NA
BMI01:TCHSIU01		3.13E−02	2	2.29E−02	1	NA	NA	NA	NA
BMI01:TRGSIU01		1.12E−02	1	NA	NA	NA	NA	NA	NA
CHOLMD021:ANTA07A		NA	NA	NA	NA	NA	NA	7.32E−03	3
CHOLMD021:ANTA07A:TCHSIU01		1.05E−03	4	NA	NA	1.04E−03	1	NA	NA
ERHA21:ANTA07A		7.04E−04	10	8.83E−04	10	7.56E−04	9	1.09E−03	4
ERHA21:APBSIU01		4.69E−06	1	1.10E−05	1	NA	NA	NA	NA
ERHA21:BMI01		2.38E−03	8	2.39E−03	10	3.17E−03	10	4.24E−03	9
ERHA21:BMI01:V1AGE01		7.31E−05	1	NA	NA	NA	NA	NA	NA
ERHA21:CENTERIDB		−3.66E−03	2	NA	NA	NA	NA	NA	NA
ERHA21:CIGT013		NA	NA	NA	NA	1.33E−03	1	NA	NA
ERHA21:INSSIU01		5.44E−05	1	NA	NA	4.75E−05	1	3.09E−05	1
ERHA21:TCHSIU01		5.37E−03	4	5.86E−03	6	9.13E−03	4	NA	NA
ERHA21:TRGSIU01		4.71E−03	4	2.57E−03	3	6.12E−03	1	NA	NA
ERHA21:V1AGE01		9.25E−04	5	1.50E−03	1	1.25E−04	1	NA	NA
ERHA21:WSTHPR01		5.09E−02	1	NA	NA	NA	NA	NA	NA
INSSIU01:CIGT012		3.32E−03	2	3.42E−03	1	NA	NA	NA	NA
INSSIU01:TCHSIU01		NA	NA	9.67E−04	1	NA	NA	NA	NA

For each cutoff fraction of occurrence in the bootstrapped CART, average coefficient and the number of times the corresponding feature is selected over the 10-fold cross validation is presented. Results are shown for medication-adjusted mean arterial blood pressure.

**Table 2:** Coefficient of determination (*R* ^2^) estimated using 10-fold cross validation of our adaptive prediction algorithm.

Method	mFDR cutoff	BP	SNP effects	cutoff fraction of occurrence							
				0.01		0.05		0.1		0.2	
Non-SNP first	0.2	adjusted	non-SNP	0.270	(0.014)	0.271	(0.014)	0.261	(0.015)	0.244	(0.015)
			main	−0.007	(0.003)	−0.009	(0.004)	−0.003	(0.003)	−0.005	(0.002)
			inter	0.001	(0.002)	−0.003	(0.004)	0.004	(0.003)	0.001	(0.002)
		unadjusted	non-SNP	0.146	(0.011)	0.141	(0.011)	0.143	(0.011)	0.130	(0.012)
			main	−0.002	(0.002)	0.004	(0.003)	0.002	(0.002)	0.000	(0.003)
			inter	−0.001	(0.003)	0.004	(0.003)	−0.007	(0.003)	−0.012	(0.005)
	0.5	adjusted	non-SNP	0.273	(0.014)	0.270	(0.014)	0.260	(0.015)	0.244	(0.014)
			main	0.220	(0.020)	0.230	(0.019)	0.232	(0.015)	0.194	(0.012)
			inter	0.248	(0.013)	0.250	(0.013)	0.238	(0.014)	0.227	(0.012)
		unadjusted	non-SNP	0.170	(0.011)	0.170	(0.011)	0.171	(0.011)	0.151	(0.013)
			main	0.128	(0.010)	0.133	(0.011)	0.128	(0.013)	0.110	(0.012)
			inter	0.170	(0.011)	0.171	(0.011)	0.172	(0.011)	0.147	(0.014)
SNP first	0.2	adjusted	SNP only	0.133	(0.010)	0.132	(0.010)	0.133	(0.010)	0.134	(0.010)
			non-SNP main	0.268	(0.013)	0.264	(0.012)	0.264	(0.012)	0.260	(0.013)
			non-SNP inter	0.271	(0.014)	0.268	(0.014)	0.261	(0.016)	0.249	(0.014)
Non-SNP first+candidate SNPs	0.2	adjusted	non-SNP	0.270	(0.014)	0.271	(0.014)	0.261	(0.015)	0.244	(0.015)
			SNP main	0.263	(0.014)	0.264	(0.014)	0.259	(0.016)	0.241	(0.016)
			SNP inter	0.269	(0.014)	0.269	(0.014)	0.261	(0.015)	0.248	(0.015)

“Non-SNP first”: the non-SNP features were first selected and the main effects of SNPs were chosen at the marginal false discovery rate cutoff of 0.2 and 0.5.

“SNP first”: the main effects of SNPs were first selected at the marginal false discovery rate cutoff of 0.2 and non-SNP effects were later included.

“candidate SNPs”: the non-SNP features were first selected and the 26 candidate SNPs were included together with the main effects of SNPs that were chosen at the marginal false discovery rate cutoff of 0.2.

For the column “BP”, “adjusted” is for results for medication-adjusted mean arterial blood pressure, and “unadjusted” for unadjusted blood pressure for each cutoff fraction of occurrence in the bootstrapped CART.

For each method and mFDR cutoff, the first row presents the baseline *R* ^2^; “main” and “inter” refers to the increase or decrease in *R* ^2^ from “non-SNP.” Standard errors of the individual *R* ^2^ for each of the ten folds are presented within parentheses.

Because prediction is better for adjusted MAP than for unadjusted MAP, plausibly a search for features predictive of MAP would yield more of them if the outcome was adjusted. Thus, plausibly, a search for features using adjusted MAP is generous approach to finding them. If a feature is not discovered when MAP is adjusted, it may have particularly limited predictive value.

### Inclusion of genome-wide SNPs as main effects did not significantly increase predictive power

Main effects of the SNPs that were chosen using the local false discovery rate machinery did not significantly improve *R* ^2^ either for medication-adjusted MAP or for unadjusted MAP (Table 2, “main”). Note that the standard errors given in Table 2 are likely to be underestimates due to correlation among the cross-validation sets. For adjusted MAP, the 10-fold cross-validation (same validation set for each fold as used in the previous subsection) of the prediction algorithm selected 49, 39, 48, and 49 SNPs in union, respectively for non-SNP cutoffs 1%, 5%, 10%, and 20%, with 18 of them common. For unadjusted MAP, the numbers were 48, 40, 48, and 68, among which 23 SNPs were shared. Between adjusted MAP and unadjusted MAP, there were 31, 22, 25, and 33 common SNPs for each of the cutoffs. No SNPs known previously to be, or thought to be, associated with SBP, DBP, and hypertension were found (see Table S3 for list of SNPs for adjusted MAP).

### Interaction effects due to SNPs may have been subsumed by the non-SNP features

Starting over the CART interaction selector, with the chosen SNPs added, also did not significantly improve *R* ^2^ (Table 2, “non-SNP first”, “mFDR = 0.2”, “inter”). At first glance, this “interaction” approach that includes gene-gene and gene-environment interactions seems slightly better than the “main effects only” approach of the previous subsection. In fact, the full procedure hardly found any SNPs: only two (rs1989858 and rs2316757, both in chromosome 17) appeared in the union of two 10-fold cross-validation experiments for adjusted and unadjusted MAP.

### Inclusion of more SNPs may diminish the quality of prediction

We raised the SNP selection cutoff (mFDR) from 0.2 to 0.5, to allow more SNPs into the adaptive regression. For adjusted MAP, inclusion of them as main effects resulted in 625, 642, 597, and 697 SNPs in union over the 10-fold cross-validation, respectively for non-SNP cutoffs 1%, 5%, 10%, and 20%, where 268 of those were common. (For unadjusted MAP, the numbers were 715, 671, 624, and 795, among which 331 were SNPs that were common to the 10 folds.) Contrary to one popular view, that use of more markers leads to improvement of predictive performance, we observed that *R* ^2^ was reduced, especially when the chosen SNPs were used as main effects (Table 2, “non-SNP first”, “mFDR = 0.5”).

### SNPs alone achieved low predictive power

For comparison, we examined the predictive power of the algorithm when SNPs were selected first and then the non-SNP features were added (See Methods). This “SNPs first” approach resulted in about 13% of *R* ^2^, compared to up to 27% of “other features first” approach under the same condition, although adding “other features” recovered the predictive power of approximately 26% (Table 2, “SNP first”). SNPs on their own seem not to have as much predictive power as other features. With both features present, the order which feature comes first does not matter.

### Candidate SNPs study

Since our unimputed SNPs did not contain SNPs whose association with blood pressure traits is believed to be known, we forced models to include these SNPs and quantified their impact on predicting MAP. We used 26 SNP that are the union of the top 10 loci for each of SBP, DBP, and hypertension traits found in Table 4 of [8]. However, this also did not improve predictive power (Table 2, “candidate SNP”). Among the 104 SNPs selected in union, 9 were from the candidate SNPs (see Table 3).

**Table 3:** List of candidate SNPs used for the analysis presented in the last set of Table 2.

SNP_rs_ID	Chr	physical_start	Gene Symbol	SBP association	DBP association	HTN association	algorithm select?
rs12046278	1	10722163		Y			Y
rs13401889	2	190618803			Y		
rs7571613	2	190513906	LOC653447	Y			
rs13423988	2	68764769					
rs17806132	2	190416531	PMS1		Y	Y	Y
rs305489	3	11986162				Y	Y
rs7640747	3	37571808	ITGA9			Y	
rs448378	3	170583592	MDS1	Y			Y
rs9815354	3	41887654	ULK4		Y		
rs899364	8	11366953				Y	Y
rs2736376	8	11155174		Y			
rs7016759	8	49574968			Y		
rs1910252	8	49569914		Y			
rs11775334	8	10109029	MSRA			Y	Y
rs1004467	10	104584496	CYP17A1	Y			
rs11014166	10	18748803	CACNB2	Y	Y	Y	
rs381815	11	16858843	PLEKHA7	Y			
rs11024074	11	16873794	PLEKHA7		Y		
rs11612893	12	129290571				Y	
rs2681472	12	88533089	ATP2B1		Y	Y	
rs2681492	12	88537219	ATP2B1	Y			Y
rs2384550	12	113837113			Y		
rs278126	12	118620099	CIT			Y	
rs3184504	12	110368990	LNK	Y	Y		Y
rs6495122	15	72912697			Y		
rs16982520	20	57192114				Y	

In columns 5–7, the entry “Y” indicated that the corresponding SNP's association with the corresponding BP trait was previously identified. The last column shows which of these SNPs were selected in our adaptive prediction algorithm.

## Discussion

Our results indicate that the inclusion of genome-wide association in addition to carefully chosen non-SNP clinical information did not result in a significant increase in the predictive power for mean arterial blood pressure. In other words, non-SNP features do as well as those with SNPs added, when the interactions among the former are exploited using the CART interaction selector. Furthermore, the more information from non-SNP features that is used, the less information SNPs add. Rather surprisingly, information from SNPs may diminish the quality of prediction. This is contrary to what has been reported from other genome-wide predictive studies [26] on type 1 diabetes (T1D), but is understandable given the noise inherent in the genome-wide data, and that T1D is relatively easy to predict. The little predictive value of genome-wide SNP data has been reported broadly [27], [28]. These studies are for predicting disease status, i.e., classification analysis, using SNPs only, while our study aims to predict a quantitative phenotype and intentionally included clinical non-SNP information, much of which is genetic. Our observations seem to support the conjecture [29]:

“… from a theoretical perspective, it can be argued that also a large number of genes will unlikely have substantial added predictive value over traditional risk factors if these variants predispose the risk factors.

“… Genetic variants may improve disease prediction beyond traditional risk factors when they are involved in unknown pathways or intermediate factors. New yet unknown pathways may be more likely for some diseases than for others.”

It appears that blood pressure traits, especially MAP, are phenotypes for which traditional risk factors confound the pathways. This is contrary to T1D, where it is believed that a genetic region with large effects (MHC) exists. Of course, it should be stressed that these results are limited to the genotyped SNPs. Perhaps including other SNPs (some causal, possibly yet to be discovered) may be more informative.

That “SNPs first” approach was not as good as “other features first” may hint at the amount of information shared by the SNPs and the non-SNP features (within the aforementioned limit). Admittedly much of the information in non-SNP clinical features is genetic. That adding the non-SNP features recovered the predictive power of the “other features first” approach may be an indication that virtually no new genetic information, or at least predictive utility, can be obtained from the markers considered in the study. As a quantitative trait, ∼13% of *R* ^2^ for “SNPs first” (and ∼26% for “other features first”) is relatively low compared to the similar figure of merits as low as 33% for mean cellular hemoglobin (MCH) for the predictive analysis of heterogeneous stock mice from eight inbred strains [15]. However, the estimated heritability of MCH in the mice population, the lowest in the study of [15], is 55%, whereas the heritability of MAP has been estimated to be roughly 33% in another population [30]. Obviously, the ARIC population is much more heterogeneous than nearly all laboratory mouse populations. This distinction may not have been emphasized sufficiently in the literature. For this reason, the main effects of SNPs found by the adaptive prediction analysis may not be very reliable.

We realize that there may be a degree of bias associated with our results. First, all of the genetic variants were chosen from the ARIC dataset and so are likely to fit the prediction set, also chosen from the same dataset, better than they might in independent samples. We consider this to be of minor concern since our focus is on the added predictive ability of genome-wide predictors, which turns out to be insignificant. Second, although our model is unconditionally highly non-linear in the data because it involves a choosing of features based on their appearances in a succession of cross-validation trees, our model is more restrictive in comparison with more general models such as generalized additive models (GAMs). Such an approach may reduce biases at the expense of added variability in the chosen model. Another possibility may be to divide MAP into intervals (e.g., low, medium, and high) much like the prediction of disease status discussed earlier. However, a major point of our paper is that we are trying to predict actual MAP, and the medical basis for such division is of question. Third, for a quantitative trait such as MAP, an adaptive spline model, which is by design smoother than CART, could be a more efficient alternative than a tree. The adaptive spline model could also suggest interactions in the first stage. We carried out an additional experiment in which CART is replaced by Multivariate Adaptive Regression Splines (MARS; see Chapter 10 of [31]) for the medication-adjusted MAP with non-SNP cutoffs 1% and 5%. For consistency, MARS was used as an interaction selector and the LASSO was fit in the same fashion as explained in the Method section. With non-SNP features only, we had cross-validated *R* ^2^ of 18.37% (standard deviation 3.58%) for cutoff 1%; 18.29% (3.56%) for cutoff 5%. With SNPs (mFDR = 0.2) added as main effects the improvement in *R* ^2^ was −0.45% (0.82%); with interaction, it was −0.38% (s.d. 1.1%). This result is similar to that of CART in that the contribution of the SNPs in predictive power was not significant. Note that MARS was only used as an interaction selector. If it subsumed the LASSO component, then MARS may have performed better than CART subsuming the LASSO, but it is unlikely that the overall predictive power be significantly higher than the best value achieved by the combined CART and LASSO approach.

We acknowledge that our predictive analysis is limited to the first-visit characteristics, and is therefore a cross-sectional study. For a predictive analysis to have a clinical utility, it would be desirable that the features can capture both averages and longitudinal changes of blood pressure. However, this entails an additional challenge. Since our study population is mature already at the first visit (45–66), we think that our cross-sectional analysis demonstrates reasonably the role of SNPs and other features in a clinically meaningful fashion.

Finally, we also acknowledge that the *R* ^2^ measure used to quantify the predictive ability of our algorithm is a population value. Particular individuals at high risk may not be identified using our method.

## Supporting Information

Table S1 **List of the ARIC clinical variables and their characteristics used as candidate non-SNP main effects for our adaptive prediction algorithm.**
(DOC)

Table S2 **Non-SNP features chosen by our adaptive prediction algorithm.** For each cutoff fraction of occurrence in the bootstrapped CART, average coefficient and the number of times the corresponding feature is selected over the 10-fold cross validation is presented. Results are shown for unadjusted mean arterial blood pressure.
(DOC)

Table S3 **SNPs chosen by our adaptive prediction algorithm.** For each cutoff fraction of occurrence in the bootstrapped CART, the union of SNPs selected over the 10-fold cross validation is presented as the entry “Y”. Results are shown for medication-adjusted mean arterial blood pressure.
(DOC)
