# Supplementary material for: SNPs and Other Features as They Predispose to Complex Disease: Genome-Wide Predictive Analysis of a Quantitative Phenotype for Hypertension
Source: PLoS One. 2011 Nov 30;6(11):e27891. doi: 10.1371/journal.pone.0027891 (PMC3227593; doi:10.1371/journal.pone.0027891)
Supplement: Table S1 — List of the ARIC clinical variables and their characteristics used as candidate non-SNP main effects for our adaptive prediction algorithm. (DOC) [file pone.0027891.s001.doc]

Supplementary Table S1

| variable | descrption | min | max | Mean | sd |
| --- | --- | --- | --- | --- | --- |
| ANTA08 | Wrist breadth (mm) | 38.00 | 62.00 | 49.83 | 3.24 |
| ANTA07A | Waist girth (cm) | 52.00 | 159.00 | 93.08 | 14.82 |
| ANTA07B | Hip girth (cm | 56.00 | 173.00 | 104.10 | 10.80 |
| ANTA07C | Calf girth (cm) | 26.00 | 58.00 | 36.71 | 3.84 |
| APASIU01 | Apolipoprotein A1 (mg/L) | 200.00 | 2690.00 | 1405.39 | 306.55 |
| APBSIU01 | Apolipoprotein B (mg/L) | 120.00 | 2370.00 | 919.04 | 286.97 |
| BMI01 | Body mass index (kg/m^2^) | 14.38 | 55.20 | 26.60 | 5.49 |
| CALC | Calcium (mg) | 76.29 | 2922.00 | 665.63 | 368.76 |
| CARB | Carbohydrate (g) | 35.28 | 598.37 | 185.74 | 75.82 |
| CHOLMD01 | Cholesterol lowering medicine use | 3.35% | | | |
| CHOLMD02 | Meds that secondarily lower cholesterol | 22.69% | | | |
| CIGT01 | Cigarette smoking status (% never) | 50.96% | | | |
| CIGTYR01 | Cigarette years of smoking | 0.00 | 3612.00 | 232.76 | 347.80 |
| DADHXCHD | Paternal history of CHD | 33.59% | | | |
| DFIB | Dietary ﬁber (g) | 0.78 | 80.23 | 17.61 | 8.14 |
| DIABTS03 | Diabetes (fasting glucose <126) | 0.07 |  |  |  |
| ERHA21 | Heart rate (per minute) | 42.00 | 130.00 | 69.08 | 9.42 |
| ETHANL03 | Usual ethanol intake (g/week) | 0.00 | 724.80 | 24.74 | 53.07 |
| EVRSMK01 | Ever smoked cigarrette? | 49.04% | | | |
| FAST0802 | Fasting time of 8 hours or more | 98.57% | | | |
| FAST1202 | Fasting time of 12 hours or more | 97.00% | | | |
| GLUSIU01 | Blood glucose level (mmol/L) | 2.05 | 23.47 | 5.71 | 1.65 |
| INSSIU01 | Insulin (pmol/L) | 7.18 | 2633.23 | 76.51 | 81.92 |
| MFAT | Monounsaturated fatty acid (g) | 1.80 | 66.48 | 20.91 | 9.35 |
| MOMHXCHD | Maternal history of CHD | 18.45% | | | |
| PFAT | Polyunsaturated fatty acid (g) | 1.32 | 40.56 | 8.46 | 3.86 |
| POTA | Potassium (mg) | 409.41 | 7416.94 | 2648.64 | 927.18 |
| PROT | Protein (g) | 13.33 | 227.19 | 68.78 | 25.71 |
| P_TFAT | Total fat (%kcal) | 8.43 | 58.77 | 32.81 | 6.73 |
| TCAL | Energy (kcal) | 511.15 | 3581.24 | 1498.32 | 522.94 |
| TCHSIU01 | Total cholesterol (mmol/L) | 1.76 | 11.56 | 5.63 | 1.07 |
| TRGSIU01 | Total triglycerides (mmol/L) | 0.29 | 12.33 | 1.42 | 0.90 |
| V1AGE01 | Age at ﬁrst visit | 44.00 | 66.00 | 54.84 | 5.84 |
| WSTHPR01 | Waist-to-hip ratio | 0.49 | 1.19 | 0.89 | 0.08 |
| CENTERID | Field center | A=36.81%, B=33.30%, D=29.89% | | | |
| HORMON02 | Hormone use (% never) | 71.77% | | | |
| MENOPS01 | Menopausal status (% postmenopausal) | 70.11% | | | |
| ANTA04 | Weight (lb) | 80.00 | 312.00 | 153.42 | 32.33 |
| HEMA09 | Fibrinogen value | 147.00 | 776.00 | 300.52 | 60.25 |
| HMTA03 | White blood count | 2.50 | 25.10 | 6.18 | 1.86 |
| HOM10D | Stroke ever diagnosed? | 0.92% | | | |
| MAGN | Magnesium (mg) | 31.27 | 719.77 | 248.62 | 88.92 |
| PRVCHD05 | Prevalent CHD | 2.01% | | | |
| P_SFAT | Saturated fatty acid (%kcal) | 2.48 | 26.77 | 12.07 | 3.04 |
