# Supplementary material for: SNPs and Other Features as They Predispose to Complex Disease: Genome-Wide Predictive Analysis of a Quantitative Phenotype for Hypertension
Source: PLoS One. 2011 Nov 30;6(11):e27891. doi: 10.1371/journal.pone.0027891 (PMC3227593; doi:10.1371/journal.pone.0027891)
Supplement: Table S2 — Non-SNP features chosen by our adaptive prediction algorithm. For each cutoff fraction of occurrence in the bootstrapped CART, average coefficient and the number of times the corresponding feature is selected over the 10-fold cross validation is presented. Results are shown for unadjusted mean arterial blood pressure. (DOC) [file pone.0027891.s002.doc]

Supplementary Table S2

| code | feature | cutoff | | | | | | | |
| --- | --- | --- | --- | --- | --- | --- | --- | --- | --- |
|  |  | 0.01 | | 0.05 | | 0.1 | | 0.2 | |
|  |  | avg coef | CV count | avg coef | CV count | avg coef | CV count | avg coef | CV count |
| **ANTA04** | Weight (lb) | 2.68E-03 | 1 | 2.23E-02 | 1 | NA | NA | NA | NA |
| ANTA07A | Waist girth (cm) | NA | NA | 3.06E-02 | 1 | 4.59E-02 | 2 | 5.57E-02 | 8 |
| APASIU01 | Apolipoprotein A1 (mg/L) | 2.34E-04 | 3 | 9.47E-04 | 1 | NA | NA | NA | NA |
| APBSIU01 | Apolipoprotein B (mg/L) | NA | NA | 1.90E-04 | 1 | 4.28E-04 | 1 | 6.33E-04 | 2 |
| BMI01 | Body mass index (kg/m2) | NA | NA | 1.49E-01 | 1 | 8.53E-02 | 2 | 2.11E-01 | 4 |
| **CALC** | Calcium (mg) | NA | NA | -5.14E-04 | 1 | NA | NA | NA | NA |
| CENTERIDB | Field center | -4.66E-01 | 7 | -9.28E-01 | 3 | -2.42E-01 | 7 | -1.93E-01 | 2 |
| CENTERIDD | **Field center** | -2.00E+00 | 10 | -1.75E+00 | 10 | -1.84E+00 | 10 | -1.87E+00 | 6 |
| CHOLMD021 | Meds that secondarily lower cholesterol | 8.27E+00 | 10 | 8.18E+00 | 10 | 8.28E+00 | 10 | 8.33E+00 | 6 |
| CIGT012 | Cigarette smoking status (% never) | NA | NA | 7.03E-01 | 3 | 7.48E-01 | 3 | 3.91E-01 | 5 |
| CIGT013 | Cigarette smoking status (% never) | 5.06E-01 | 4 | 4.43E-01 | 3 | 8.83E-01 | 7 | 7.08E-01 | 9 |
| CIGTYR01 | Cigarette years of smoking | -5.19E-04 | 8 | -2.99E-04 | 9 | -5.38E-04 | 8 | -5.37E-04 | 6 |
| **ERHA21** | Heart rate (per minute) | NA | NA | NA | NA | NA | NA | 1.42E-01 | 4 |
| ETHANL03 | Usual ethanol intake (g/week) | 6.81E-03 | 8 | 4.81E-03 | 6 | 5.38E-03 | 7 | 6.27E-03 | 2 |
| **GLUSIU01** | Blood glucose level (mmol/L) | NA | NA | -2.87E-02 | 1 | NA | NA | NA | NA |
| INSSIU01 | Insulin (pmol/L) | NA | NA | NA | NA | 1.24E-04 | 1 | 2.88E-03 | 5 |
| TCHSIU01 | Total cholesterol (mmol/L) | NA | NA | NA | NA | 4.46E-01 | 2 | 5.93E-01 | 10 |
| TRGSIU01 | Total triglycerides (mmol/L) | 3.35E-01 | 8 | 2.41E-01 | 10 | 3.32E-01 | 10 | 5.29E-01 | 9 |
| V1AGE01 | Age at ﬁrst visit | 3.69E-02 | 2 | 7.77E-02 | 6 | 1.10E-01 | 9 | 1.42E-01 | 10 |
| WSTHPR01 | Waist-to-hip ratio | 2.41E+00 | 5 | 9.30E-01 | 2 | 2.31E+00 | 5 | 4.34E+00 | 4 |
| ANTA07A:TCHSIU01 | | 7.32E-03 | 2 | 4.70E-03 | 2 | 3.68E-04 | 1 | NA | NA |
| BMI01:CIGT012 | | 4.94E-02 | 3 | 2.42E-03 | 1 | 1.60E-02 | 1 | NA | NA |
| BMI01:CIGT013 | | 5.21E-02 | 3 | 2.92E-02 | 1 | 3.81E-02 | 1 | NA | NA |
| BMI01:ERHA21 | | NA | NA | NA | NA | 5.42E-03 | 1 | 4.62E-03 | 1 |
| BMI01:TCHSIU01 | | 1.75E-02 | 5 | 2.35E-02 | 4 | 2.34E-02 | 2 | NA | NA |
| BMI01:V1AGE01 | | 2.75E-03 | 4 | 2.69E-03 | 4 | 4.27E-03 | 2 | NA | NA |
| CIGT012:ANTA07A | | 6.93E-03 | 1 | 4.06E-03 | 1 | 7.29E-03 | 1 | NA | NA |
| CIGT012:GLUSIU01 | | 4.43E-02 | 2 | 5.30E-03 | 1 | 3.30E-03 | 1 | NA | NA |
| CIGT012:TCHSIU01 | | 1.68E-02 | 1 | NA | NA | NA | NA | NA | NA |
| CIGT013:ANTA07A | | 1.15E-03 | 1 | NA | NA | NA | NA | NA | NA |
| CIGT013:TCHSIU01 | | 2.83E-01 | 2 | 2.17E-01 | 1 | 2.86E-01 | 1 | NA | NA |
| CIGT013:TRGSIU01 | | 4.36E-01 | 1 | NA | NA | NA | NA | NA | NA |
| ERHA21:ANTA07A | | 7.10E-04 | 8 | 7.52E-04 | 10 | 9.85E-04 | 8 | 5.89E-04 | 3 |
| ERHA21:ANTA07A:TCHSIU01 | | 1.08E-05 | 1 | 6.52E-05 | 1 | NA | NA | NA | NA |
| ERHA21:ANTA07A:V1AGE01 | | 2.51E-05 | 1 | NA | NA | NA | NA | NA | NA |
| ERHA21:APBSIU01 | | 9.46E-06 | 3 | NA | NA | NA | NA | NA | NA |
| ERHA21:BMI01 | | 3.58E-04 | 1 | 1.14E-03 | 4 | 2.29E-03 | 6 | 4.35E-03 | 5 |
| ERHA21:BMI01:TCHSIU01 | | 1.37E-04 | 2 | 4.43E-04 | 1 | NA | NA | NA | NA |
| ERHA21:BMI01:V1AGE01 | | 5.76E-05 | 4 | 7.46E-05 | 1 | NA | NA | NA | NA |
| ERHA21:CENTERIDB | | -1.03E-02 | 1 | NA | NA | NA | NA | NA | NA |
| ERHA21:CIGT013 | | 1.05E-02 | 2 | 8.61E-03 | 5 | NA | NA | NA | NA |
| ERHA21:INSSIU01 | | NA | NA | NA | NA | NA | NA | 1.57E-05 | 1 |
| ERHA21:INSSIU01:CIGT012 | | 7.61E-05 | 1 | NA | NA | NA | NA | NA | NA |
| ERHA21:INSSIU01:TCHSIU01 | | 1.19E-05 | 1 | 1.42E-05 | 1 | NA | NA | NA | NA |
| ERHA21:TCHSIU01 | | 3.66E-03 | 3 | 6.57E-03 | 3 | 8.71E-03 | 7 | NA | NA |
| ERHA21:TRGSIU01 | | 3.46E-03 | 1 | NA | NA | NA | NA | NA | NA |
| ERHA21:V1AGE01 | | 9.09E-04 | 9 | 8.15E-04 | 8 | 7.11E-04 | 3 | NA | NA |
| ERHA21:WSTHPR01 | | 2.70E-02 | 3 | 1.42E-01 | 1 | NA | NA | NA | NA |
| GLUSIU01:CIGT012 | | 1.56E-01 | 1 | 1.47E-01 | 1 | 1.10E-01 | 1 | NA | NA |
| INSSIU01:BMI01 | | NA | NA | NA | NA | NA | NA | 1.25E-04 | 1 |
| INSSIU01:CIGT012 | | 3.40E-03 | 5 | 3.32E-03 | 1 | 3.74E-03 | 2 | NA | NA |
| INSSIU01:TCHSIU01 | | 1.76E-04 | 8 | 1.88E-04 | 7 | 4.05E-04 | 8 | NA | NA |
| TCHSIU01:ANTA07A | | 3.56E-03 | 3 | 4.77E-03 | 1 | 5.81E-04 | 1 | NA | NA |
| TCHSIU01:BMI01 | | 2.73E-02 | 1 | 2.83E-02 | 1 | NA | NA | NA | NA |
| TCHSIU01:CIGT012 | | 7.44E-02 | 1 | 7.01E-02 | 1 | NA | NA | NA | NA |
| TCHSIU01:CIGT013 | | 4.05E-01 | 1 | 5.79E-01 | 1 | 2.77E-01 | 1 | NA | NA |
