# Supplementary material for: SNPs and Other Features as They Predispose to Complex Disease: Genome-Wide Predictive Analysis of a Quantitative Phenotype for Hypertension
Source: PLoS One. 2011 Nov 30;6(11):e27891. doi: 10.1371/journal.pone.0027891 (PMC3227593; doi:10.1371/journal.pone.0027891)
Supplement: Table S3 — SNPs chosen by our adaptive prediction algorithm. For each cutoff fraction of occurrence in the bootstrapped CART, the union of SNPs selected over the 10-fold cross validation is presented as the entry “Y”. Results are shown for medication-adjusted mean arterial blood pressure. (DOC) [file pone.0027891.s003.doc]

Supplementary Table S3

| SNP_rs_ID | Chr | physical_start | Gene.Symbol | cutoff01 | cutoff05 | cutoff10 | cutoff20 |
| --- | --- | --- | --- | --- | --- | --- | --- |
| rs6728372 | chr2 | 56753058 |  |  |  | Y | Y |
| rs11465670 | chr2 | 102400871 | IL18RAP | Y | Y | Y | Y |
| rs1345198 | chr2 | 106784467 |  |  |  | Y | Y |
| rs1347104 | chr3 | 14775422 | C3orf20 |  |  | Y |  |
| rs2742395 | chr3 | 45733598 | SACM1L | Y |  |  |  |
| rs1962800 | chr3 | 45754946 | SACM1L | Y | Y | Y | Y |
| rs267120 | chr3 | 76858286 |  | Y |  |  |  |
| rs12631158 | chr3 | 102060120 | ABI3BP | Y | Y | Y |  |
| rs17307478 | chr6 | 24713002 | KIAA0319 | Y | Y | Y |  |
| rs2651206 | chr6 | 43321454 | TTBK1 | Y | Y |  |  |
| rs1096699 | chr6 | 43636418 | XPO5 |  | Y |  |  |
| rs13206405 | chr6 | 73720534 | KCNQ5 | Y | Y |  |  |
| rs1321162 | chr6 | 76513066 |  | Y | Y | Y | Y |
| rs885582 | chr6 | 82668915 |  | Y | Y | Y | Y |
| rs2875087 | chr6 | 82672801 |  | Y | Y | Y |  |
| rs209405 | chr6 | 83600447 |  | Y | Y |  | Y |
| rs2282123 | chr6 | 89964279 | GABRR1 | Y | Y | Y | Y |
| rs846960 | chr6 | 108073621 | FLJ10159 | Y | Y |  |  |
| rs846963 | chr6 | 108075562 | FLJ10159 | Y | Y | Y | Y |
| rs598549 | chr6 | 147545491 | LOC645818 |  |  | Y |  |
| rs6931675 | chr6 | 149426860 | UST | Y |  |  |  |
| rs7742644 | chr6 | 149467988 |  | Y |  |  |  |
| rs1521193 | chr8 | 9933428 |  |  |  | Y |  |
| rs11249969 | chr8 | 9969387 | MSRA | Y |  | Y |  |
| rs814424 | chr8 | 9980333 | MSRA | Y |  |  |  |
| rs1052453 | chr8 | 10145734 | MSRA |  | Y |  |  |
| rs545556 | chr9 | 77285777 |  |  |  | Y |  |
| rs7048294 | chr9 | 88609666 |  |  |  |  | Y |
| rs7044513 | chr9 | 89417268 | DAPK1 |  |  | Y |  |
| rs2808374 | chr9 | 109026899 |  |  |  | Y | Y |
| rs7851038 | chr9 | 111388181 |  |  |  | Y |  |
| rs4937782 | chr11 | 132874541 | OPCML |  |  | Y |  |
| rs7108751 | chr11 | 132895943 | OPCML |  | Y | Y |  |
| rs12799484 | chr11 | 132904709 | OPCML | Y | Y | Y | Y |
| rs474863 | chr13 | 45958363 |  |  | Y | Y |  |
| rs9316206 | chr13 | 45984579 |  |  |  |  | Y |
| rs6561313 | chr13 | 45996897 |  |  | Y | Y | Y |
| rs4942674 | chr13 | 47190845 |  |  | Y | Y |  |
| rs1036450 | chr13 | 57463101 |  |  | Y |  |  |
| rs10444649 | chr13 | 57506409 |  |  |  | Y | Y |
| rs10149796 | chr14 | 33343320 |  |  |  |  | Y |
| rs1951273 | chr14 | 34876719 |  | Y |  |  |  |
| rs7157104 | chr14 | 35068136 |  |  |  |  | Y |
| rs11621835 | chr14 | 55402744 |  |  |  |  | Y |
| rs3783848 | chr14 | 88927477 | CHES1 | Y |  |  | Y |
| rs12884621 | chr14 | 95132929 |  |  |  |  | Y |
| rs2604972 | chr14 | 97450710 |  |  |  |  | Y |
| rs4905631 | chr14 | 97469917 |  | Y |  |  |  |
| rs7161307 | chr14 | 97677435 |  | Y |  |  |  |
| rs12883126 | chr14 | 100203760 | C14orf70 |  | Y |  |  |
| rs11631967 | chr15 | 34356320 |  |  |  |  | Y |
| rs2959930 | chr15 | 70385667 | BRUNOL6 | Y |  |  |  |
| rs17643123 | chr15 | 89687451 |  | Y | Y | Y | Y |
| rs391578 | chr16 | 76552595 | KIAA1576 |  |  |  | Y |
| rs11079742 | chr17 | 42375126 |  |  |  | Y |  |
| rs2316757 | chr17 | 42421223 |  | Y | Y | Y | Y |
| rs3851798 | chr17 | 42421265 |  |  | Y |  |  |
| rs11085020 | chr19 | 3397106 | NFIC | Y | Y | Y | Y |
| rs3859530 | chr19 | 33231120 |  | Y |  | Y |  |
| rs12984120 | chr19 | 33241849 |  | Y |  | Y |  |
| rs1171096 | chr19 | 53225879 | CABP5 | Y |  | Y | Y |
| rs10418719 | chr19 | 53228536 | CABP5 | Y |  |  |  |
| rs10415779 | chr19 | 55431885 | MYH14 | Y |  |  |  |
| rs584975 | chr19 | 55433381 | MYH14 | Y |  |  |  |
| rs6140046 | chr20 | 662612 |  |  |  |  | Y |
| rs199300 | chr20 | 15108751 | C20orf133 |  |  |  | Y |
| rs6110544 | chr20 | 15114774 | C20orf133 | Y | Y | Y | Y |
| rs200759 | chr20 | 15554420 | C20orf133 | Y | Y |  | Y |
| rs6080112 | chr20 | 15994323 |  |  |  |  | Y |
| rs8125763 | chr20 | 17831530 |  |  |  |  | Y |
| rs7267772 | chr20 | 21482969 |  |  | Y |  |  |
| rs804605 | chr20 | 22140056 |  |  |  |  | Y |
| rs804606 | chr20 | 22140445 |  |  |  |  | Y |
| rs6048704 | chr20 | 23244208 |  |  |  | Y | Y |
| rs6059100 | chr20 | 31181355 |  | Y | Y | Y | Y |
| rs6087449 | chr20 | 31184290 |  |  | Y |  | Y |
| rs6030239 | chr20 | 40500012 | PTPRT |  |  | Y |  |
| rs6102925 | chr20 | 40661591 | PTPRT | Y | Y | Y | Y |
| rs8121443 | chr20 | 40667589 | PTPRT | Y | Y | Y | Y |
| rs6102967 | chr20 | 40702734 | PTPRT | Y | Y | Y | Y |
| rs6073142 | chr20 | 41660425 | IFT52 | Y | Y | Y | Y |
| rs393115 | chr20 | 41708912 | IFT52 |  |  | Y |  |
| rs6091653 | chr20 | 51307118 | ZNF218 |  |  | Y | Y |
| rs17339050 | chr20 | 52772668 |  | Y |  |  | Y |
| rs7273633 | chr20 | 59214877 |  | Y | Y | Y | Y |
| rs6093073 | chr20 | 59235860 |  | Y | Y | Y |  |
| rs2295001 | chr20 | 61011472 | DIDO1 | Y | Y |  | Y |
| rs9612192 | chr22 | 21668069 |  |  |  |  | Y |
| rs9614202 | chr22 | 42855970 | PARVB | Y |  | Y |  |
| rs2267617 | chr22 | 42870020 | PARVB | Y |  |  |  |
| rs2267620 | chr22 | 42870625 | PARVB | Y | Y | Y | Y |
| rs5767834 | chr22 | 46389361 |  | Y |  | Y | Y |
